# The evaluation and treatment of postintubation tracheal strictures

**DOI:** 10.7196/AJTCCM.2018.v24i2.214

**Published:** 2018-06-21

**Authors:** Ivan Schewitz

**Affiliations:** Department of Cardio-thoracic Surgery, University of Pretoria, and Linksfield Park Clinic, Linksfield, Johannesburg, South Africa

## Editorial

Tracheal strictures following prolonged ventilation have been well described for at least 50 years. The condition can be successfully treated with resection and end-to-end anastomosis. The use of low-pressure endotracheal cuffs, better attention to nursing detail and advances in invasive ventilation techniques have markedly decreased the incidence. However, strictures following intubation are unfortunately still observed. The paper by Perumal *et al*. ^[1]^ in this issue of the *AJTCCM* relates the South African experience for a condition that is becoming rare.

In a developing country, where many patients are treated by non-specialists, this paper is important as it highlights a potentially life-threatening complication associated with invasive ventilation of a patient. Given the high incidence of trauma experienced in the country, patients may receive suboptimal treatment, especially in rural areas where specialists are not available.

The misdiagnosis of asthma in a patient who has been ventilated needs to be stressed, as a stricture can develop after a surprisingly short period of ventilation. Suspected tracheal strictures should be referred to a centre of expertise as a matter of urgency. Symptoms develop only after the diameter of the trachea has reduced by 50%, and are an indication of a significant stricture.

The paper is a timely reminder of a potentially life-threatening complication of invasive ventilation, which is seen too often in developing countries and which should be eliminated with good medical and nursing care.
